# LncRNAs as Therapeutic Targets and Potential Biomarkers for Lipid-Related Diseases

**DOI:** 10.3389/fphar.2021.729745

**Published:** 2021-08-04

**Authors:** Shi-Feng Huang, Xiao-Fei Peng, Lianggui Jiang, Ching Yuan Hu, Wen-Chu Ye

**Affiliations:** ^1^ The Sixth Affiliated Hospital of Guangzhou Medical University, Qingyuan People’s Hospital, Qingyuan, China; ^2^ Department of Human Nutrition, Food and Animal Sciences, College of Tropical Agriculture and Human Resources, University of Hawaii at Manoa, Honolulu, HI, United States

**Keywords:** lncRNAs, therapeutic targets, lipid metabolism, lipid-related diseases, biomarkers

## Abstract

Lipid metabolism is an essential biological process involved in nutrient adjustment, hormone regulation, and lipid homeostasis. An irregular lifestyle and long-term nutrient overload can cause lipid-related diseases, including atherosclerosis, myocardial infarction (MI), obesity, and fatty liver diseases. Thus, novel tools for efficient diagnosis and treatment of dysfunctional lipid metabolism are urgently required. Furthermore, it is known that lncRNAs based regulation like sponging microRNAs (miRNAs) or serving as a reservoir for microRNAs play an essential role in the progression of lipid-related diseases. Accordingly, a better understanding of the regulatory roles of lncRNAs in lipid-related diseases would provide the basis for identifying potential biomarkers and therapeutic targets for lipid-related diseases. This review highlighted the latest advances on the potential biomarkers of lncRNAs in lipid-related diseases and summarised current knowledge on dysregulated lncRNAs and their potential molecular mechanisms. We have also provided novel insights into the underlying mechanisms of lncRNAs which might serve as potential biomarkers and therapeutic targets for lipid-related diseases. The information presented here may be useful for designing future studies and advancing investigations of lncRNAs as biomarkers for diagnosis, prognosis, and therapy of lipid-related diseases.

## Introduction

Lipid metabolism is an intricate and complex physiological process that is involved in the progression of lipid-related diseases (Li et al., 2017). Importantly, since modern society is associated with irregular lifestyle patterns and long-term nutrient overload, severe lipid metabolism disorders and lipid accumulation have become commonplace (Liu and Ding, 2017; Dłubek et al., 2021). Abnormal lipid metabolism is the primary feature of several refractory chronic diseases (Yang et al., 2016), such as atherosclerotic disease (Michos et al., 2019), obesity (Wang et al., 2014), fatty liver disease (Vernon et al., 2011), and diabetes mellitus (Garde et al., 2019). Thus, developing novel tools and strategies for maintaining cholesterol homeostasis is urgently required to prevent and treat these diseases.

Long non-coding RNAs (lncRNAs) are a class of RNA that do not encode proteins (Kim et al., 2009). Instead, they are involved in complex biological processes and pathophysiological conditions, including lipid metabolism disorders (Zeng et al., 2018; Simion et al., 2019). Recently, numerous clinical studies have shown that lncRNAs impair cholesterol homeostasis and play a critical role in the progression of lipid-related diseases (Han et al., 2019; Ou et al., 2020). For example, a primate-specific lncRNA (*CHROME*) was found to be elevated in the plasma and atherosclerotic plaques of patients with coronary heart disease (CHD) (Hennessy et al., 2019). Similarly, highly up-regulated in liver cancer (*HULC*) lncRNA was discovered to modulate the deregulation of lipid metabolism in hepatoma cells and result in malignant development (Cui et al., 2015). These findings suggest that lncRNAs regulate lipid metabolism and promote the development of lipid-related diseases. LncRNAs might also function as the miRNAs sponges and affect lipid metabolism and related diseases (Lan et al., 2019). Importantly, lncRNAs also play an essential in the progression of some other diseases, such as cancer (Hahne and Valeri, 2018). Much research has been conducted on the specific functions of lncRNAs in these diseases.

The emerging role of lncRNAs as potential biomarkers and therapeutic targets for lipid-related diseases has not explicitly been summarised, and the present review aims to fill this gap in the literature. LncRNAs have been increasingly recognized as potential biomarkers for various human diseases, including atherosclerosis (Simion et al., 2020), MI (Spiroski et al., 2021), liver disease (Yang et al., 2021), and cancer (Xing et al., 2021). Here, we mainly reviewed the recent investigations of the role of lncRNAs as potential biomarkers and therapeutic targets in lipid-related diseases. Findings from this review would summarize the mechanisms by which lncRNAs act as biomarkers and therapeutic targets for lipid-related diseases.

## LncRNAs Mechanisms of Action

**FIGURE 1:**
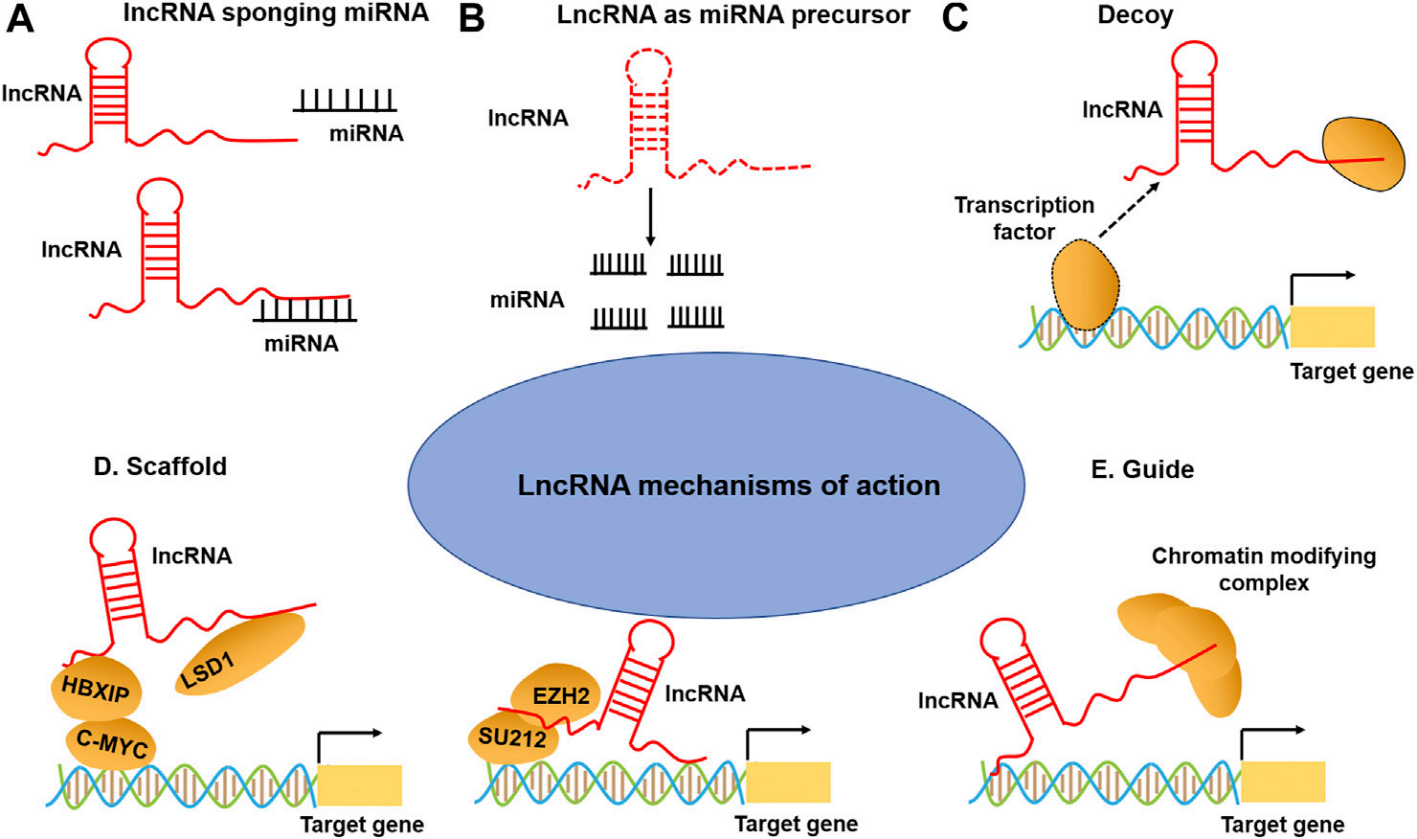
LncRNA mechanisms of action. **(A)** LncRNAs can act as a sponge to titrate miRNAs away from their mRNA targets. **(B)** The lncRNAs can act as miRNA precursors. **(C)** LncRNA can bind to transcription factors or other proteins as a decoy and sequester them away from chromatin (lower-right). **(D)** LncRNA can also serve as a scaffold to promote the assembling of chromatin remodeling complexes. **(E)** LncRNA can guide transcription factors to specific genomic locations for regulating gene expression.

Recent studies have illustrated that lncRNAs can bind to the proteins, RNA, DNA, or a combination of them to exert their functions (Fasolo et al., 2019; Hu Y. et al., 2019). As regulators of gene expression, lncRNAs involve in various biological processes (Fernandes et al., 2019; Mumtaz and Online, 2017), acting as miRNA sponge, decoys, scaffolds, guides, and post-translation regulation (Rinn and Chang, 2012) (Figure 1). For instance, many lncRNAs act as a miRNA sponge to regulate miRNAs and their targets. For example, small nucleolar RNA host gene 16 (*SNHG16*) facilitated the development and progression of neuroblastoma by upregulating homeobox A7 (*HOXA7*) expression via sponging miR-128–3p (Bao et al., 2020). Decoying lncRNAs mediated transcriptional repression by guiding chromatin modifiers such as m^6^A formation and recognition to genomic targets, such as *XIST* (Patil et al., 2016), *HOTAIR* (Loewen et al., 2014), and *GAS5* (Sun et al., 2017). LncRNAs can be used as scaffolds to form enhancer loops or as structural components of ribonucleoprotein complexes (Stackhouse et al., 2020). Nuclear paraspeckle assembly transcript 1 (*NEAT1*) scaffolds broadly interacts with NONO/PSF and other RNA-binding proteins (RBPs) and that globally enhance pri-miRNA processing (Jiang et al., 2017). Additionally, many lncRNAs exert their functions by sequestering regulatory factors in the nucleus or cytoplasm: for example, colon cancer-associated transcript-2 (*CCAT2*) can block miR-145 maturation by inhibiting pre-miR-145 export to cytoplasm (Yu Y. et al., 2017); whereas cytoplasmic lncRNAs, such as *lincRNA-p21*, interact with RNA-binding protein HuR to recruit let-7/Ago2 to inhibit their repression of *lincRNA-p21* stability (Yoon et al., 2012). Finally, lncRNAs can act as enhancers or co-activators of target gene activation, such as *H19* and *GAS5*. LncRNA may have more than one function, varying by subcellular localization, stimuli, and/or cell types. With the continuous increase of lncRNA-mediated functions, it has become clear that they are important regulators of multiple biological and cellular processes and can be used as candidate diagnostic and prognostic biomarkers for human diseases.

## LncRNAs Participate in the Development of Lipid-Related Diseases

**FIGURE 2:**
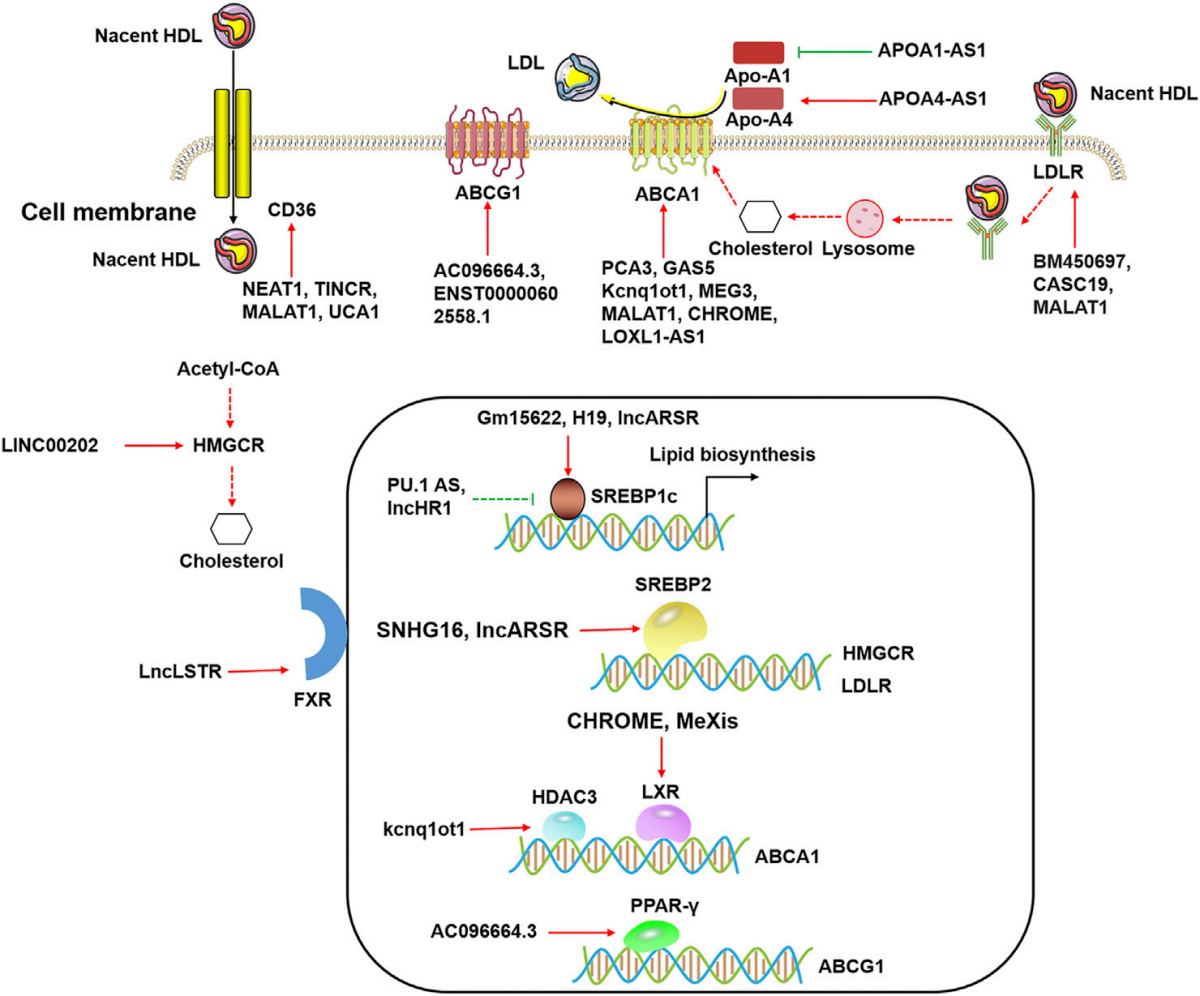
The functional roles of lncRNAs in lipid metabolism. LncRNAs modulate cholesterol efflux by ABCA1, ABCG1, CD36, and LDLR in the cytoplasm. LncRNAs regulate ABCA1 expression by HDAC3 and LXRs, and lncRNAs regulate ABCG1 expression by PPAR-γ in the nucleus. LncRNAs influence lipid biosynthesis by SREBP1c and SREBP2 in the nucleus.

Lipid metabolism is the biosynthesis and biodegradation of lipids in cells (Santos and Schulze, 2012). It involves the breakdown and storage of fats for energy and the synthesis of structural and functional lipids (de Carvalho and Caramujo, 2018). Lipid biosynthesis is a part of metabolic abnormalities in cells, which require large quantities of lipids to synthesize cytomembranes, organelles, and signaling molecules during cell proliferation (Xu et al., 2020). Importantly, fatty acid oxidation (FAO) can provide abundant ATP for cells (Jeon et al., 2012), and fatty acids are a major source of ATP molecules (Fhu and Ali, 2020). In addition, lncRNAs affect gene expression that is involved in lipid metabolism (Table 1). Numerous studies have shown that lncRNAs participate in lipid metabolism by influencing the expression of key genes, networks, and pathways involved in lipid biosynthesis, cholesterol transport, lipid uptake, and cholesterol efflux (Figure 2).

**TABLE 1:** Summary of the act of lncRNAs as therapeutic targets and potential biomarkers for lipid-related diseases.

LncRNAs	Dys-regulation	Human samples	Targets	Molecular mechanisms	Diseases	References
*HOXC-AS1*	Up	Carotid atherosclerosis	HOXC6	Facilitates HOXC6 expression	Atherosclerosis	Huang et al. (2016)
*GAS5*	Down	Atherosclerotic plaque	—	—	Atherosclerosis	Chen et al. (2017)
*RAPIA*	Up	Atherosclerotic plaque	miR-183-5p, ITGB1	Promotes ITGB1 expression by targeting miR-183-5p	Atherosclerosis	Sun et al. (2020)
*MIAT*	Up	Serum	miR-149-5p, CD47	Promotes CD47 expression by targeting miR-149-5p	Atherosclerosis	Ye et al. (2019)
*LncRNA-ATB*	Up	Serum	Caspase-3	Promotes the expression of caspase-3	Atherosclerosis	Yu et al. (2019)
*CHROME*	Up	Plasma	miR-27b, miR-33a, miR-33b, miR-128 and ABCA1	Regulates cholesterol efflux and nascent HDL particle formation by miRNAs/ABCA1 pathway	Atherosclerosis	Hennessy et al. (2019)
*RP11-714G18.1*	Down	Atherosclerotic plaques	LRP2BP, MMP1	Display athero-protective role via LRP2BP/MMP1 pathway	—	—
*CASC11*	Down	Plasma	IL-9	Improve atherosclerosis by inhibiting IL-9 expression	Atherosclerosis	Tao et al. (2019)
*NEXN-AS1*	Down	Atherosclerotic plaques, blood	NEXN	Mitigates atherosclerosis by regulating NEXN	CAD	Hu et al. (2019a)
*ENST00000416361*	Up	Plasma	SREBP1, SREBP2	Promotes SREBP1 and SREBP2 expression	CAD	Li et al. (2020a)
*MEG3*	Up	Tissues	miR-26a, Smad1	Promotes Smad1 expression by targeting miR-26a	CAD	Bai et al. (2019)
*ANRIL*	Up	Tissue	EZR, CXCL11 or TMEM106B	Exerts opposing effects on endothelial cell activities associated with coronary artery disease	CAD	Cho et al. (2020)
*Ang362*	Up	Plasma	—	—	CHD	Wang et al. (2020a)
*KCNQ1OT1*	Up	Serum	miR-26a-5p, ATG12	Promotes cardiomyocyte autophagy and aggravates MI by miR-26a-5p/ATG12 axis	MI	Li et al. (2021a)
*LINC00261*	Up	Tissues	miR-522-3p, TNRC6A	Promotes MI through the miR-522-3p/TNRC6A axis	MI	Jiang et al. (2021)
*NRF*	Up	Blood	—	—	MI patients with HF	Yan et al. (2020)
*NEAT1*	Up	Blood	miR-378a-3p, ATG12	Promotes cardiomyocytes injury by targeting miR-378a-3p	MI	Zhao et al. (2020)
*CHAST*	Up	Blood	—	—	MI	Wang et al. (2020b)
*MALAT1*	Up	Tissue	miR-144-3p	Promotes cardiomyocyte apoptosis after MI via targeting miR-144-3p	MI	Gong et al. (2019)
*TTTY15*	Up	Blood	miR-455-5p, JDP2	Promotes hypoxia-induced cardiomyocytes injury by targeting miR-455-5p	MI	Huang et al. (2019a)
*CAIF*	Down	Tissues and serum	—	—	MI	Wu et al. (2019)
*MALAT1*	Up	Serum	miR-200a-3p, PDCD4	Regulates cardiomyocytes apoptosis after via modulating miR-200a-3p/PDCD4 axis	MI	Sun and Zhang, (2019)
*TUG1*	Up	Aortic valves	miR-204-5p, Runx2	Promotes osteoblast differentiation by miR-204-5p/Runx2 axis	CAVD	Yu et al. (2018)
*LncARSR*	Up	Serum	SREBP-2, HMGCR	Increases SREBP-2 expression and HMGCR.	Hypercholesterolemia	Huang et al. (2018)
*HULC*	Up	HCC tissues	ASCL1, PPARA	miR-9/PPARA/ACSL1/cholesterol/RXRA/HULC signalling	Hepatocellular carcinoma	Cui et al. (2015)
*NEAT1*	Up	Serum	miR-129-5p, SOCS2	Promotes liver fibrosis by miR-129-5p/SOCS2	ASH	Ye et al. (2020)
*MALAT1*	Up	Liver biopsy	miR-20b-5p, TXNIP	Promotes TXNIP expression by targeting mR-20b-5p	NAFLD	Li et al. (2021b)
*LeXis*	Up	Liver biopsy	—	—	NAFLD	Park et al. (2020)
*B4GALT1-AS1*	Down	Liver tissues	hnRNPA1	Recruits hnRNPA1 to suppress hepatic lipogenesis and gluconeogenesis	NAFLD	Wang et al. (2018)
*GAS5*	Up	Plasma	—	—	NAFLD	Han et al. (2020)
*LncARSR*	Up	Liver tissues	Akt, SREBP-1c	Promotes hepatic lipogenesis via Akt/SREBP-1c pathway	NAFLD	Zhang et al. (2018)
*Lnc18q22.2*	Up	Liver tissues	—	—	NAFLD	Atanasovska et al. (2017)
*RP11-142A22.4*	Up	Visceral adipose tissue	miR-587, Wnt5β	Promotes adipogenesis by sponging miR-587 to modulate Wnt5β expression	Obesity	Zhang et al. (2020c)
*LINC00473*	Down	Adipose tissue	—	—	Obesity and type-2 diabetes	Tran et al. (2020)
*E330013P06*	Up	Blood	—	—	Breast cancer patient with type-2 diabetes	Chen et al. (2020)
*SNHG8*	Up	Blood	SOCS3, ICAM1	Promotes SOCS3 or ICAM1 expression by sponging miR-411-5p	AMI	Zhuo et al. (2019)

Recent studies have reported that lncRNAs participate in the regulation of various genes expression in lipid metabolism that was induced by hormones (Fu et al., 2020), environmental stress (Wen et al., 2020), lipid/cholesterol (Ma et al., 2018), and obesity/type 2 diabetes (Hu et al., 2020). A single lncRNA often targets multiple mRNAs, and these mRNAs are linked to the different metabolic pathways (Huang, 2018). It is important to note that each mRNA is typically targeted by several lncRNAs, enabling coordinated gene expression. Many molecules are involved in lipid metabolism, including nuclear transcription factors such as LXR, FXR, SREBP, and the scavenger receptor CD36 (Figure 2) (Shimano and Sato, 2017; Yan et al., 2018; Piccinin et al., 2021). These regulatory molecules, along with lncRNAs, are implicated in the regulation of lipid metabolism.

Given the fact that lipid metabolism is distributed different cellular organelles also transport of the intermediates between the different organelles is an important point in lipid metabolism (Khor et al., 2013; Xu and Taubert, 2021). Fox example, lipid metabolism is located in the endoplasmic reticulum (ER) for lipid biosynthesis (Jacquemyn et al., 2017), mitochondria and peroxisomes for β-oxidation (Zhou et al., 2018), lipid droplets (LDs) for storage and transport (Freyre et al., 2019), and lysosomes for lipid hydrolysis and recycling (Go et al., 2012). Lipid metabolism includes processes such as lipid uptake, biosynthesis, catabolism, and secretion. LncRNAs can affect biological functions in many ways, such as the miRNA sponge, guide or decoy, scaffold, and chromatin remodeling. Currently, numerous lncRNAs have been identified to be involved in the regulation of lipid metabolism. However, many lncRNAs with lipid metabolism functions do not directly target genes involved in lipid metabolism pathways (He et al., 2019; Lan et al., 2019), such as triglyceride and cholesterol biosynthesis and fatty acid oxidation. Instead, they target the lncRNA-miRNA-mRNA and lncRNA-mRNA axes. For example, the lncRNA *HULC* has been shown to regulate abnormal lipid metabolism by decreasing miR-9 expression, leading to the upregulation of RXRA expression (Cui et al., 2015). RXRA, a member of the RXR family that can be activated by sterol (Costet et al., 2000), modulates the lipid metabolism disorders by activating acyl-CoA synthetase long-chain family member 1 (ACSL1) (Cui et al., 2015). Similarly, lncRNA *PU.1 AS* regulates lipid metabolism via the sterol regulatory element-binding protein-1c (SREBP-1c) pathway, resulting in reduced triglyceride synthesis (Dong et al., 2019). Transcription factors of the SREBP family, including SREBP-1a, SREBP-1c, and SREBP-2, are central to transcriptional control of genes related to lipid and fatty acid metabolism (Brown and Goldstein, 1999). Interestingly, overexpression of SREBP-1c is known to facilitate fatty acid and triglyceride synthesis and lead to lipid accumulation in the liver (Yan et al., 2016). On the other hand, the inhibition of SREBP-1c is shown to alleviate lipid accumulation and lipotoxicity (Jin et al., 2020). The involvement of a lncRNA derived from hepatocytes (*lnc-HC*) in lipid metabolism has been extensively reported. For example, *lnc-HC* was found to regulate PPARγ-mediated lipid metabolism and triglyceride (TG) concentration via miR-130b-3p, where *lnc-HC* expression was positively correlated with the miR-130b-3p expression (Lan et al., 2019). Furthermore, it has been illustrated that *lnc-HC* forms a complex with hnRNPA2B1 and negatively regulates Cyp7a1 and Abca1 expressions; both are implicated in hepatocytic cholesterol metabolism (Lan et al., 2016). Another lncRNA and hnRNP complex has also been identified with LeXis and RALY hnRNP, which are involved in lipid metabolism and influence metabolic gene expression (Sallam et al., 2016).

## Diseases Associated With lncRNA-Related Lipid Dysregulation

Several diseases, including atherosclerosis, MI, liver disease, and hypercholesterolemia, are caused by or associated with lipid dysregulation (Butt et al., 2017; Gluchowski et al., 2017; Michos et al., 2019). Importantly, studies focused on these diseases were performed using patient specimens, animal models (ApoE−/− and LDL−/−), and atherosclerosis model cell lines, such as human umbilical vein endothelial cells (HUVECs) (Chen L. et al., 2019), human peripheral blood monocytes (THP-1) (Choi et al., 2021), human vascular smooth muscle cells (HVSMCs) (Li X. et al., 2021). Therefore, we only summarised several representative studies that mainly focused on lncRNA functions in lipid-related disease processes.

Disruption of lipid metabolism has been confirmed as a significant factor in the pathogenesis of atherosclerosis (Sukhorukov et al., 2020). The progression of atherosclerosis is known to be regulated by disturbances of lipid metabolism (Lovren et al., 2015), which impairs endothelial cells’ function. Recent studies have identified *H19* as a well-known lncRNA associated with atherosclerosis (Huang Y. et al., 2019). *H19* expression has been reported to be up-regulated in patients with atherosclerosis and may be a potential therapeutic target for atherosclerosis (Yang Y. et al., 2019). Knockdown of *H19* inhibits hyperlipidemia and alleviates atherosclerotic lesions in HFD-treated ApoE−/− mice (Pan and sciences, 2017; Shi et al., 2020), while lentivirus-mediated H19-forced expression increase the plaque area size (Huang Y. et al., 2019). Technically, *H19* acts as a molecular sponge for miR-148b-3p and activates its expression of ELF5 (E74 like ETS transcription factor 5), resulting in the restoration of ELF5 that inhibit the cell migration in ox-LDL-stimulated HUVECs (Liu S. et al., 2021). Additionally, *lncARSR*, a lncRNA regulator of Akt signaling associated with HCC and RCC, has recently been studied as a potential therapeutic target for cholesterol disorder, and its downstream target SREBP-2 was identified. SREBP-2 has been found to bind to HMG-CoA reductase (HMGCR) to promote hepatic cholesterol biosynthesis, resulting in aberrant regulation of cholesterol metabolism (Huang et al., 2018). Collectively, lncARSR-SREBP-2-HMGCR plays a pivotal role in regulating lipid metabolism and the development of atherosclerosis (Xiao and Song, 2013).

Dysregulated lipid metabolism is a hallmark of non-alcoholic steatohepatitis (NASH), a very common liver disorder (Musso et al., 2013). Recently, growing evidence has suggested that dysregulated lncRNA expression is associated with inflammation and fibrosis in NASH (Leti et al., 2017). Whole transcriptome analysis and identified differentially expressed lncRNAs (*RP11-128N14.5* and *TGFB2-OT1*) in patients with non-alcoholic fatty liver disease (NAFLD) (Di Mauro et al., 2019). Several lncRNAs, including hepatocellular carcinoma up-regulated lncRNA, *NEAT1*, and metastasis-associated lung adenocarcinoma transcript 1 (*MALAT1*), were highly expressed in liver biopsies from NAFLD patients (Leti et al., 2017). Furthermore, expression of *MALAT1* was upregulated in livers of ob/ob mice and hepatocytes exposed to palmitate (Yan et al., 2016). Another lncRNA, Alu*-*mediated p21 transcriptional regulator (APTR), was discovered to be significantly increased in human cirrhosis and activate hepatic stellate cells (Yu et al., 2015). Hepatic *LeXis* expression is a mediator of cholesterol biosynthesis (Sallam et al., 2016). Thus, raising or lowering *LeXis* levels influence the expression of genes involved in cholesterol biosynthesis and alter liver and plasma cholesterol levels (Sallam et al., 2016). Brown fat-enriched lncRNA 1 (*Blnc1*) was strongly elevated in obesity and NAFLD in mice (Zhao et al., 2018). Hepatic Blnc1 deficiency is suggested to abrogate high-fat diet-induced hepatic steatosis and insulin resistance and ameliorate NASH pathogenesis (Zhao et al., 2018). These findings provide a further rationale for analyzing global changes in lncRNA expression in NAFLD and NASH.

**FIGURE 3:**
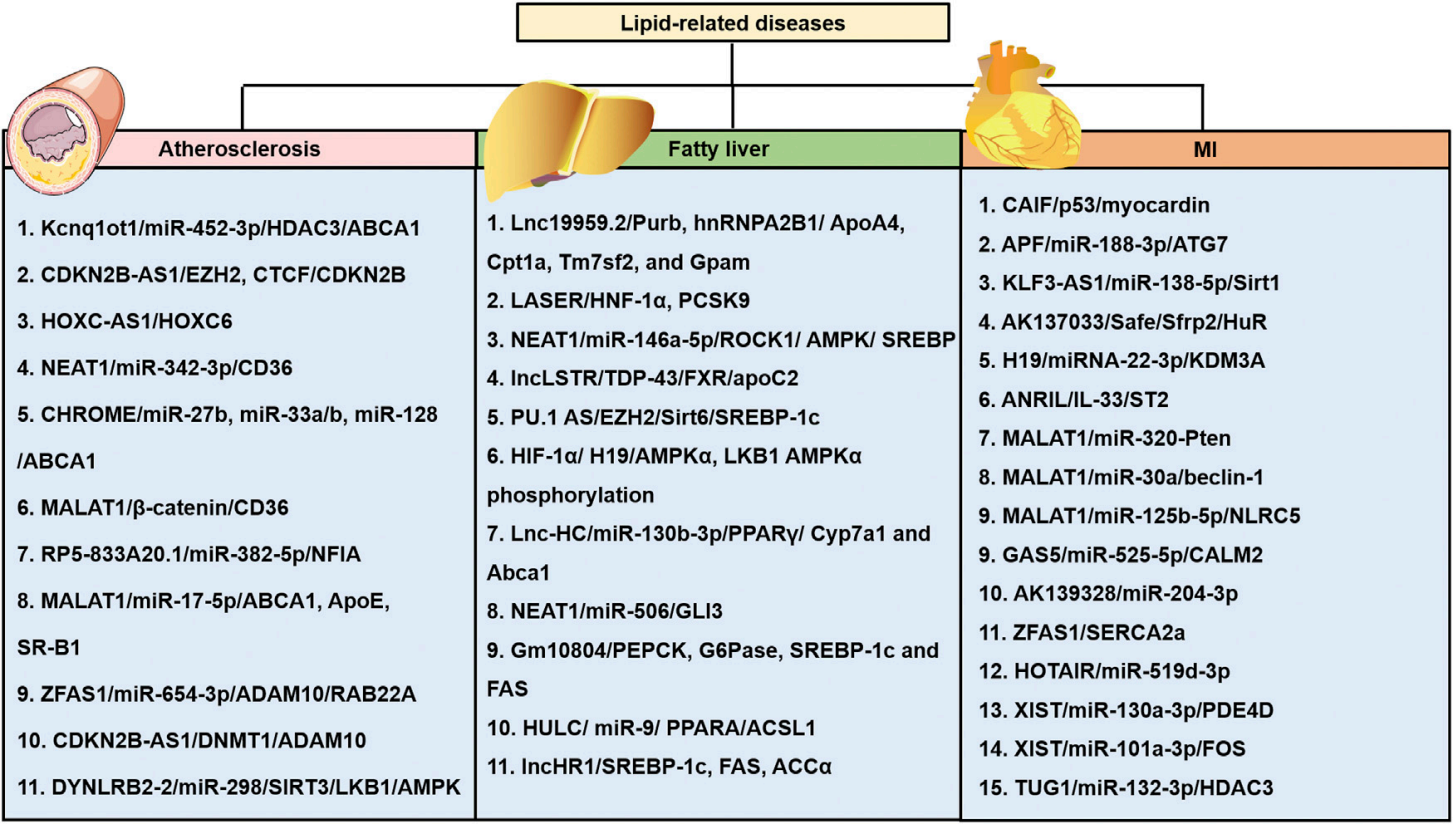
LncRNAs are involved in the three major diseases, including atherosclerosis, NAFLD, and myocardial infarction (MI) caused by abnormal cholesterol levels and various lipid fractions. Various lncRNAs and their mechanisms are illustrated. APF: autophagy promoting factor; CAIF: cardiac autophagy inhibitory factor; CALM2: calmodulin 2; GSA5: growth arrest-specific transcript 5; lncRNA XIST: long non-coding RNA X-inactive specific transcript; NLRC5: nucleotide-binding and oligomerization domain-like receptor C5; Sfrp2: secreted frizzled-related protein 2.

Recent bioinformatics and high-throughput sequencing studies have revealed that lncRNAs are differentially expressed in patients with hypoalphalipoproteinemia and MI caused by abnormal lipid metabolism (Wang et al., 2019). Differently expressed lncRNAs and mRNAs in atherosclerosis by analyzing dataset GSE28829 (Wang et al., 2019). A total of 654 lncRNAs and 5,784 mRNAs were significantly dysregulated in the progression of atherosclerosis (Wang et al., 2019). Moreover, six lncRNAs, *ZFAS1* (ZNFX1 antisense RNA 1), *LOC100506730*, *LOC100506691*, *DOCK9-AS2*, *RP11-6I2.3*, and *LOC100130219*, were confirmed as potential novel therapeutic and prognostic targets for atherosclerosis (Wang et al., 2019). LncRNA *ENST00000416361* was higher in the plasma of 50 patients with coronary artery disease (CAD) than the 50 healthy volunteers (Li P. et al., 2020). SREBP1 and SREBP2 were also up-regulated in CAD patients and showed positive correlations with ENST00000416361 (Li P. et al., 2020). Single nucleotide polymorphisms (SNPs) on the cyclin-dependent kinase inhibitor 2B antisense RNA (*ANRIL*) and *MALAT1*, two lncRNAs, affect the prognosis of MI (Li Y. et al., 2020). *ANRIL* rs9632884 and *MALAT1* rs3200401 were significantly associated with the lipid levels of both controls and MI patients (Li Y. et al., 2020). KCNQ1 overlapping transcript 1 (*KCNQ1OT1*) was found to be increased in the serum of myocardial infarction (MI) patients, ischemia/reperfusion (I/R) mouse and hypoxia/reoxygenation (H/R)-induced cell model (Li J. et al., 2021). Moreover, several SNPs interacted with sex and age and modified the total cholesterol (rs9632884), LDL-C (rs1537373), and creatinine levels, affecting the risk of MI (Li Y. et al., 2020). These studies using clinical specimens and *in vitro* disease models have suggested that lncRNAs are involved in lipid-related diseases. However, the results should be further validated via *in vitro* and *in vivo* systems. Further research is required to analyze potential biomarkers and therapeutic targets in various lipid-related diseases (see Figure 3). This review provides a comprehensive insight into the current knowledge regarding the involvement of lncRNAs in regulating lipid metabolism, which may unveil the potential biomarkers and therapeutic targets for treating lipid-related diseases (Table 1).

## LncRNAs are Ideal Diagnostic Biomarkers and Therapeutic Targets

Diagnosis of several lipid-related diseases and their associated disease risks are mainly accomplished by analyzing the concentrations of lipid components such as total cholesterol, HDL, LDL, and triglycerides in the blood (Gotto, 2011; Paredes et al., 2019). This method only obtains accurate results when patients are fasted for at least 9–12 h. However, it provides limited information on cholesterol levels. Thus, it is necessary to search for better diagnostics and novel biomarkers for lipid-related diseases to overcome these disadvantages. LncRNAs are present in body fluids and are as stable as mRNA. Due to their tissue-specific properties, lncRNAs can be used as clinical indicators for diagnosis and are expected to become a new target for disease treatment (Table 1). Therefore, the application of lncRNAs as diagnostic biomarkers can result in a timely collection of more accurate and detailed disease information and risk factor data.

Previous attempts to use lncRNAs as biomarkers for disease diagnosis have been demonstrated in several cancer studies (Ratti et al., 2020) (Table 2). They revealed the functional roles of lncRNAs during cancer progression, including tumorigenesis, metastasis, and resistance to cancer treatment (Shen et al., 2015; Bin et al., 2018). Interestingly, some lipid-related lncRNAs mentioned in this review have also been emphasized in some cancer studies and proposed as potential diagnostic biomarkers (Peng et al., 2020). For example, the *CHROME*, which is mainly involved in cholesterol efflux and HDL biogenesis, was elevated in the plasma and atherosclerotic plaques of individuals and identified as a novel biomarker for the progression of CAD (Hennessy et al., 2019). On the other hand, plasma *LeXis*, which participates in cholesterol metabolism and the development of hepatic steatosis, was found to act as a non-invasive diagnostic biomarker for NASH (Park et al., 2020). *NEAT1* and *ANRIL*, which are associated with cholesterol synthesis and MI, respectively, were suggested to be biomarkers that identify non-small cell lung carcinoma (NSCLC) (Yu X. et al., 2017; Osielska and Jagodziński, 2018). Furthermore, elevated plasma levels of *HULC*, which is involved in cholesterol synthesis, were identified as a biomarker for liver cancer (Xie et al., 2013). Additionally, the correlation between *MALAT1*, known to participate in cholesterol efflux, and lung cancer has been suggested as a diagnostic indicator (Lin et al., 2018). Moreover, the role of *TUG1*, an atherosclerosis-associated lncRNA, in various cancers has been previously studied (Niu et al., 2017; Guo et al., 2019). *TUG1* was found to recruit specific RNA-binding proteins to facilitate cancer progression (Duan et al., 2019). These results suggest that lncRNAs play multiple functional roles in various disease processes and, as has frequently been reported in recent studies, cholesterol homeostasis is closely related to cancer occurrence. Collectively, these reports on lncRNAs in cancer indicate that the development of lncRNA biomarkers for diagnosing lipid-related diseases is very promising.

**TABLE 2:** Summary of data from relevant lncRNAs-based biomarkers in human multiple tumors.

Biomarkers	Dys-regulation	Tumors	Sample type	Sample size	Technological approach	Application	Comments	References
*LncRNA-ATB FAM83H-AS1*	Up	Breast cancer	Serum	90 breast cancer patients	RT-PCR	Prognosis; disease monitoring	Serum lncRNA-ATB and FAM83H-AS1 could be used as a non-invasive diagnostic marker for early stages of breast cancer	El-Ashmawy et al. (2020)
*LINC00114, LINC00261*, *HOTAIR*	HOTAIR (Up), LINC00114 and LINC00261 (Down)	CRC	Tissues	459 nonmetastatic CRC samples and 87 metastatic CRC samples	RT-PCR	Prognosis; disease monitoring	3-lncRNA signature that includes LINC00114, LINC00261, and HOTAIR is an independent factor for predicting CRC prognosis	Liu et al. (2020)
*MSC-AS1*	Up	LC	Tissues	123 LC patients (111 tumor	—	—	—	—
tissues, 12 adjacent normal samples)	RT-PCR	Diagnosis and prognosis	MSC-AS1 may be used as a potential biomarker of LC.	Liu et al. (2021b)	—	—	—	—
*HELIS LINC01093, CYTOR*	HELIS and LINC01093 (Down), CYTOR (Up)	HCC	Tissues	82 paired tissue samples from patients with HCC	RT-PCR	Prognosis; disease monitoring	Down-regulated HELIS and LINC01093, up-regulated CYTOR are perspectives for differential diagnostics of HCC	Burenina et al. (2021)
*SNHG18*	Up	HCC	Tissues, Plasma	71 paired HCC patients	RT-PCR	Diagnosis	—	—
*DLG2-AS1*	Down	LUAD	Tissues	70 LUAD patients	RT-PCR	Prognosis; disease monitoring	DLG2-AS1 serves as a good diagnostic biomarker for LUAD patients	Arenas et al. (2020)
*MIAT, LINC00460, and LINC00443*	MIAT and LINC00460 (Up) LINC00443 (Down)	KIRC	Tissues	530 KIRC patients	RT-PCR	Prognosis; disease monitoring	The LPM based on three-lncRNAs could serve as independent prognostic factors with a tremendous predictive ability for KIRC patients	Zhang et al. (2020a)
*SAMMSON*	Up	OSCC, GBM	Tissues, Plasma	90 OSCC patients	—	—	—	—
56 patients with GBM (34 males and 22 females)	RT-PCR	Diagnosis and prognosis	SAMMSON might play a critical role in OSCC progression and serve as a novel prognostic and diagnostic biomarker in OSCC.	—	—	—	—	—
Plasma SAMMSON has diagnostic value for GBM	Xie et al. (2019); Zheng et al. (2020)	—	—	—	—	—	—	—
*LUCAT1*	Up	PTC	Tissues	61 PTC patients	RT-PCR	Diagnosis and prognosis	LUCAT1 can act as a novel prognostic biomarker for patients with PTC	Luzón-Toro et al. (2019)
*PTENP1*	Down	BC	Plasma	50 patients with BC and 60 healthy controls	RT-PCR	Diagnosis	Exosomal PTENP1 is a potential novel biomarker that can be used for the clinical detection of BC.	Zheng et al. (2018)
*PANDAR, FOXD2-AS1, SMARCC2*	Up	GC	Plasma	109 GC patients and 106 healthy controls	RT-PCR	Diagnosis	Plasma PANDAR, FOXD2-AS1, and SMARCC2 may be appropriate diagnostic biomarkers for GC.	Yang et al. (2019b)

Importantly, from a therapeutic perspective, the best approach to prevent and treat lipid-related diseases is to make certain lifestyle modifications, such as exercising more and consuming a healthy diet (Mannu et al., 2013). However, if high lipid levels persist, medication must be taken to lower them. As mentioned earlier, the diagnosis criteria for lipid-related diseases are based on detecting cholesterol levels present in plasma (Płaczkowska et al., 2014). Thus, the primary purpose of treatment is to reduce cholesterol to appropriate levels. However, it is essential to note that the relationship between cholesterol and lipid-related diseases is ever-changing, which means that treatments also vary depending on the type and condition of the related disease. For instance, statin-based drugs, bile acid sequestrants, and cholesterol absorption inhibitors (Ezetimibe) are used clinically for different conditions. Specifically, statins decrease substances required for liver cholesterol production, bile oxides or bile acid sequestrants facilitate bile acid production from cholesterol, and cholesterol absorption inhibitors reduce cholesterol and limit cholesterol absorption from the small intestine (Taoufiq et al., 2011). In addition, drugs that only increase the absorption of LDL cholesterol have also been increasingly used recently (Lee et al., 2020). Due to their specific actions and side effects, these drugs are commonly used in combination in clinical and surgical treatments.

Importantly, lncRNAs involved in lipid metabolism can also be used as potential therapeutic targets to maintain cholesterol levels in the normal range. In general, RNA interference (RNAi), using shRNA, siRNA, or anti-sense oligonucleotide (ASO), is the most promising approach to target lncRNA silencing (Chi et al., 2017). This approach has been proven effective at the whole animal and cellular levels through various research (Liu et al., 2017; Zhang L. et al., 2020). For instance, the lentiviral shRNA targeting of lncRNA myocardial infarction associated transcript (*MIAT*) significantly attenuates atherosclerosis progression and increases plaque stability *in vivo* (Ye et al., 2019). Thus, a novel method for achieving safe and efficient RNAi delivery should be investigated and developed by further research. Furthermore, ASO-based methods are also studied for more stable and less off-target occurrence in addition to RNA interference technology (Maruyama and Yokota, 2020). For example, *MALAT1* targeted ASO has been developed, and its inhibitory effect has been identified using animal models of malignancy (Amodio et al., 2018). Moreover, besides the method that targets lncRNA itself, controlling lncRNA function by inhibiting its interaction with the RNA-binding proteins has also been attempted (Kung et al., 2013; Bhat et al., 2016). However, note that RNA interference therapeutics have recently been progressed through preclinical development into clinical trials (Bobbin and Rossi, 2016). Thus, applying these as ideal clinical therapeutics requires the development of safe and effective delivery systems.

Small molecules have been extensively used for the therapeutic targeting of various diseases. These compounds have greater cellular uptake and fewer administrative challenges than antisense oligonucleotides and viral vectors for RNAi delivery. Small molecule inhibitors target lncRNAs by preventing them from binding to their RNA-binding proteins (RBPs). After analysing the lncRNA expression profiles from lncRNA modulator atlas in pan-cancer (LncMAP) database by bioinformatics analysis, the lncRNA network consists of 1,206 nodes and 4,770 drug-lncRNA associations to examine the global relationship between small molecule drugs and their affected lncRNAs (He et al., 2019). In addition, small molecules were screened to modulate the lncRNA HOX transcript antisense RNA (*HOTAIR*)-enhancer of zeste homolog2 (EZH2) interaction using alphaScreen technology (Pedram Fatemi et al., 2015). The interaction was inhibited with HOTAIR-polycomb repressive complex 2 (PRC2) binding through small-molecule intervention resulting in reduced metastatic phenotypes in many cancers, including breast (Gupta et al., 2010), colorectal (Kogo et al., 2011), and hepatocellular carcinomas (El-Khazragy et al., 2020). However, it is necessary to investigate the lncRNA-protein interaction and pharmacological trends further to develop more effective small molecule drugs (Figure 3).

## Conclusion and Future Perspectives

Recent studies have shown that lncRNAs are involved in various lipid-related diseases (Table 1), thereby opening up a new research field and providing insight for lncRNAs as important eukaryotic transcripts. Concerning the correlation between lncRNAs regulation and lipid-related diseases, atherosclerosis is the most frequently studied disease (Ye et al., 2021). The occurrence of lipid-related diseases is due to the inactivation of suppressor genes and the activation of pathogenic genes. Thus, screening and identifying candidate biomarkers for prognosis, monitoring, and evaluating patients’ responses to therapies is required to develop novel strategies for lipid-related disease therapies. Also, ncRNAs (miRNAs and lncRNAs), DNA methylation, and histone modifications can epigenetically regulate gene expression. LncRNAs have recently served as important regulators of lipid-related diseases via various biological processes, including lipid metabolism, lipid accumulation, lipid synthesis, and cholesterol efflux (Sallam et al., 2018; Chen X. et al., 2019; Wang Z. et al., 2020; Zuo et al., 2020). Thus, there is a considerable thrill in using lncRNAs as a critical therapeutic target in treating lipid-related diseases.

Recent studies have demonstrated that lncRNAs could be detected in the blood plasma, tumor tissue, and urine, making them serve as promising biomarkers for development as disease, including atherosclerosis, MI, and cancer diseases (Dastmalchi et al., 2020; Fattahi et al., 2020). Genome-wide sequencing techniques have emerged as an important technology and reported a large number of newly dysregulated lncRNAs, implying promising results about the broad application prospects of lncRNAs in the prognosis and diagnosis of lipid-related diseases. Deregulation of many lncRNAs, such as *H19* (Pan, 2017), *TUG1* (Li et al., 2018), *GAS5* (Chen et al., 2017), *RAPIA* (Sun et al., 2020), *MIAT* (Ye et al., 2019), *CASC11* (Tao et al., 2019), *NEXN-AS1* (Hu Y.-W. et al., 2019), and *lnc00113* (Yao et al., 2018), has been detected in patients with atherosclerosis. LncRNAs including *H19*, *TUG1*, *MIAT*, and *CASC11* could be detected in serum samples as a potential diagnostic marker in patients with atherosclerosis. In addition to establishing the functional role of lncRNAs in diagnosis, some lncRNAs such as *AL117190.1*, *COL4A2-AS1*, *LINC00184*, *MEG3* and *MIR22HG* could function as crucial prognostic markers for patients (Yao et al., 2019). Besides, as diagnostic and prognostic markers, lncRNAs such as *H19* (Yörüker et al., 2018), *MEG3* (Wan and Zhao, 2020), *PVT1* (Pan et al., 2019), FAM83H antisense RNA 1 (*FAM83H-AS1*) (El-Ashmawy et al., 2020), *SNHG1* (Xiao et al., 2018), and *LUCAT1* (Xing et al., 2021) are involved in the process of various cancer progression. Thus, we speculate that dysregulated lncRNAs may be used as biomarkers to provide diagnosis and prognostic of lipid-related diseases but also are useful in therapeutic applications.

Although it is well established that high concentrations of serum cholesterol levels facilitate the development of atherosclerosis (Johnston et al., 2017), the association of LDL-C or other lipids with atherosclerosis remains controversial. To date, a large number of lncRNAs associated with lipid metabolism and lipid-related diseases have been identified through RNA-seq and bioinformatics analyses. The functions of these lncRNAs may have important clinical implications in lipid metabolism and lipid-related diseases since they provide a myriad of possibilities for the diagnostics and treatment of these diseases. Furthermore, lncRNAs have been described as high tissue- and cell type-specific expression patterns (Kopp and Mendell, 2018; Antonov et al., 2019), which could be classified as different subclasses of lipid-related diseases or even predict responses to treatments. However, our current knowledge of the effect of lncRNAs on lipid-related diseases is possibly only the tip of the iceberg. Thus, more comprehensive investigations should be conducted to better understand how lncRNAs affect lipid-related diseases and develop new therapies.

The study of lncRNAs involved in controlling the cholesterol levels, specifically lncRNAs that directly interact with target genes or epigenetic proteins at the transcriptional level, may contribute to developing novel drugs to treat lipid-related diseases. Importantly, the latest next-generation sequencing-based big data research has identified numerous lncRNAs associated with various lipid-related diseases (Ye et al., 2021). However, further molecular biological research is needed to deepen the understanding of the association between various lncRNAs discovered and actual genetic mechanisms.

**FIGURE 4:**
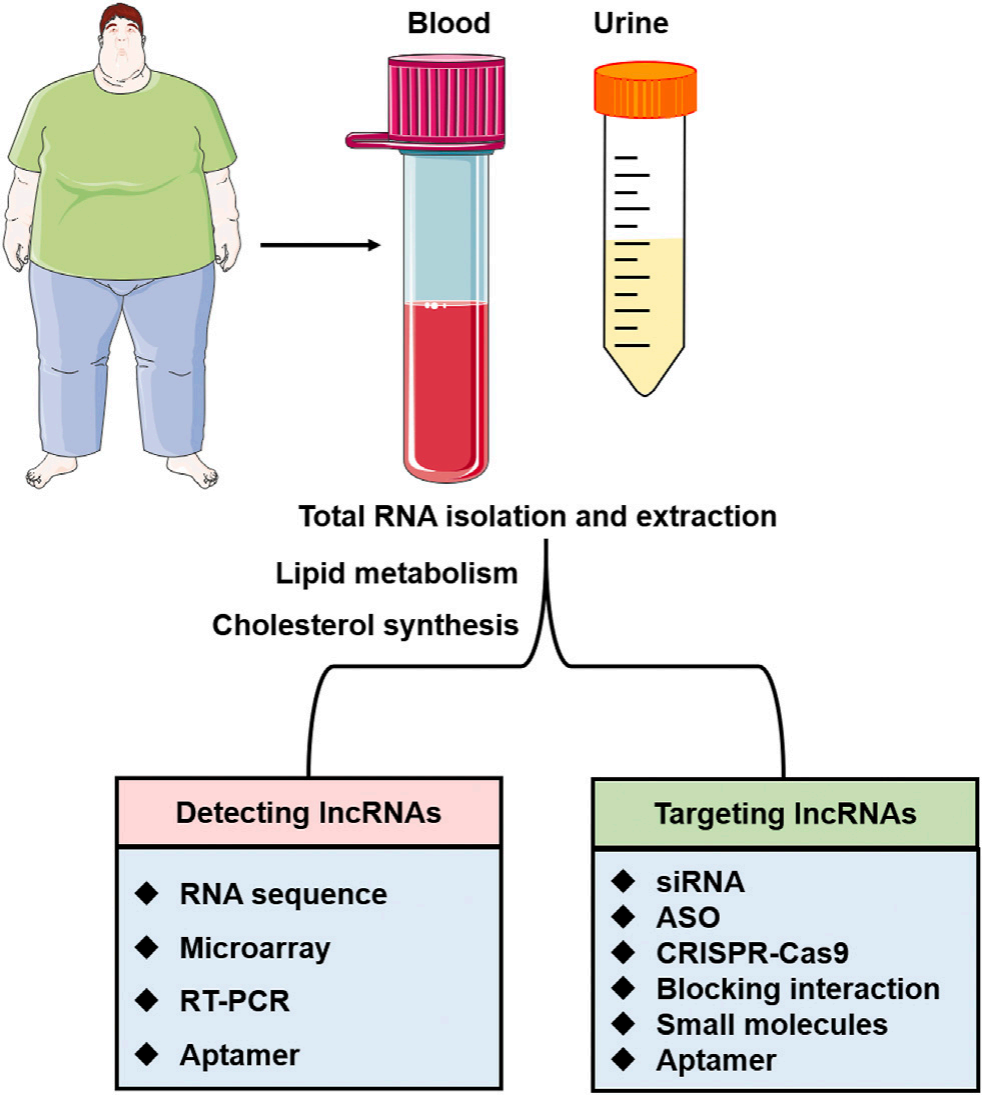
The application of lncRNAs as therapeutic targets and diagnostic biomarkers. LncRNAs in urine or blood specimens can be detected by various methods such as RNA sequence, microarray, RT-PCR, and aptamer. The interactions of lncRNAs with target proteins and lncRNAs involved in lipid metabolism and cholesterol synthesis will be the potential therapeutic targets for lipid-related diseases.

This review summarised various lipid-related lncRNAs and their target genes that play essential roles in lipid metabolism and lipid-related diseases. The involvement of lncRNAs was abnormally expressed in certain disease conditions, including atherosclerosis (Gao and Guo, 2021), myocardial infarction (Li J. et al., 2021), non-alcoholic fatty liver disease (Li J.-z. et al., 2021), and hypercholesterolemia (Tontonoz et al., 2017). Furthermore, a large number of lncRNAs identified from various studies were found to be associated with a diverse range of diseases. As lncRNAs are structurally and functionally conserved, further research is required to develop more effective diagnostics and therapeutics in this field or reveal the mechanism of certain diseases (see Figure 4 and Table 1). Altogether, advancing the knowledge of these lncRNAs and their functions is crucial for developing novel detection and modification methods.

## Glossary

AMI: Acute myocardial infarction
ASH: Alcoholic steatohepatitis
ATG12: autophagy-related 12 homologs
CAIF: Cardiac autophagy inhibitory factor
CASIMO1: Cancer-associated small integral membrane open reading frame 1
CAVD: calcific aortic valve disease
CHAST: cardiac hypertrophy-associated transcript
HF: heart failure
HMGCR: HMG-CoA reductase
HOXC6: homeobox C6
HOXC-AS1: lncRNA HOXC cluster antisense RNA 1
ITGB1: integrin β1
JDP2: Jun dimerization protein 2
LDLR: low-density lipoprotein receptor
LRP2BP: low-density lipoprotein related receptor 2 binding protein
MALAT1: metastasis-associated lung adenocarcinoma transcript 1
MIAT: myocardial infarction associated transcript
MMP1: matrix metalloproteinase 1
NEXN: nexilin F-actin binding protein
NEXN-AS1: nexilin F-actin binding protein antisense RNA 1
PDCD4: programmed cell death 4
PPARA: proliferator-activated receptor alpha
RAPIA: associated with the progression and intervention of atherosclerosis
SOCS2: cytokine signalling 2
SQLE: squalene epoxidase
TNRC6A: trinucleotide repeat-containing gene 6a
BC: bladder cancer
CYTOR: cytoskeleton regulator RNA
FAM83H-AS1: FAM83H antisense RNA 1
FOXD2-AS1: FOXD2 adjacent opposite strand RNA 1
GBM: glioblastoma
GC: gastric cancer
HCC: hepatocellular carcinoma
KIRC: kidney renal clear cell carcinoma
LC: laryngeal cancer
LncRNA-ATB: lncRNA activated by TGF β
CRC: colorectal cancer
LPM: lncRNA prognostic model
LUAD: lung adenocarcinoma
MSC-AS1: MSC antisense RNA 1
OSCC: oral squamous cell carcinoma
PANDAR: promoter of CDKN1A antisense DNA damage activated RNA
PCa: prostate cancer
PTC: papillary thyroid cancer
PTENP1: phosphatase and tensin homolog pseudogene 1
SAMMSON: survival associated mitochondrial melanoma-specific oncogenic non-coding RNA
SMARCC2: SWI/SNF related, matrix associated, actin-dependent regulator of chromatin subfamily c member 2
SNHG18: small nucleolar RNA host gene 18
